# Pattern of mental ill health morbidities following hysterectomy for benign gynaecological disorders among Nigerian women

**DOI:** 10.1186/1752-4458-3-18

**Published:** 2009-07-30

**Authors:** Michael A Okunlola, Celestine Umuerri, Olayinka O Omigbodun, Imran O Morhason-Bello, Stella N Okonkwo, Oladosu A Ojengbede

**Affiliations:** 1 Department of Obstetrics and Gynaecology, University College Hospital, Ibadan, Nigeria; 2 Department of Psychiatry, University College Hospital, Ibadan, Nigeria

## Abstract

**Objective:**

to compare the pre and post hysterectomy mental ill health (MIH) status and also, to determine whether there is any association with the surgical indication.

**Methodology:**

An observational study, conducted among women scheduled for hysterectomy at the University College Hospital, Ibadan from January till June 2005. The MIH morbidities were assessed using a validated general health questionnaire (GHQ) before and after the surgery by trained research assistant. The score of 4 and above was used as the cut off. Cross tabulations were performed to detect any association and also to compare pre and post hysterectomy mental health status. The level of statistical significance was set at P < 0.05.

**Results:**

Of the 50 women recruited, 45 participated in the study. The age range of the participants was 35 to 63 years with a mean of 48.6 (SD = 0.6) years. Anxiety related disorder was present in 20 (44.4%), and depression in 3 (6.7%) before hysterectomy. Post surgery, there was significant increase in those with anxiety by 6.8% and a reduction in the proportion of depressive illness by 2.3%. Uterine fibroid as a preoperative diagnosis, had significant association among those with anxiety related disorder (68.4%) and depression (10.5%).

**Conclusion:**

This study suggests that mental ill health may complicates hysterectomy for benign uterine pathology among Nigerian women, and that anxiety related disorders increases after operation with the highest proportion in those with clinical diagnosis of Uterine Fibroid. We recommend adequate preoperative counseling using properly trained psychologists when affordable to minimize these morbidities.

## Background

Surgical patients have long been reported with varying psychosomatic symptoms before and after major operation [1-3]. The degree of their manifestations is influenced by quality of preoperative information provided, the clinical diagnosis of whether benign or malignant, associated postoperative complications as well as extent of re-integration into the routine premorbid activities1[1,4,5]. Furthermore, Clinicians especially in developed countries have included "feelings of general wellbeing" as an integral aspect of quality of care offered aside the surgical intervention. This evidence has necessitated the inclusion of psychologists into the mainstream of management protocol[4].

Hysterectomy, the surgical removal of the uterus, is one of the most commonly performed gynaecological operations in the world. In USA for example, 60 to 70 percent of women are at risk of hysterectomy in their life time[6], and about 40 percent among the Australian population. The extent of surgery depends on the clinical diagnosis. Simple hysterectomy is usually performed for benign conditions while radical hysterectomy is reserved for suspected malignant diagnosis. The commonest indication is benign uterine diagnosis such as uterine fibroid, dysfunctional uterine bleeding, endometriosis and chronic pelvic pain[7].

There are concerns about the reported association of mental ill health following hysterectomy for benign conditions [8-10]. The patterns reported were increased anxiety state, depressive illness, mood changes, and acute psychotic illness[10,11]. This association was sometimes flawed by lack of strict patient selection - as most of the women were perimenopausal with no details of whether they were on hormone replacement therapy (HRT) or not, exclusion of possible risk factors such as drug abuse, prior psychiatry illness, poorly standardized tool to measure mental illness morbidities, and the observation that majority of the studies were retrospective in design[2,12,13]. On the contrary, recent prospective studies revealed paradoxical findings, as majority show an improvement in pattern of mental ill health[12,14]. This observed variation was adjudged to be due to exclusion of possible risk factors and other confounders such as HRT use[13,15-17].

In the African setting, womanhood is believed to be strongly linked to procreation. Therefore, preservation of their womb is jealously guarded, and the womb is usually believed to be their symbol of identity. Inspite of this, hysterectomy still remains the commonest gynaecological major operation in many health care facilities in Nigeria, and the pattern of indication is comparable to that in developed countries[7]. Although, there is no National data on the prevalence of hysterectomy among Nigerian women but, hospital based data showed a range of 7.5 to 25 percent of gynaecological surgeries[18,19]. Morbidities usually reported are those directly related to surgical mishap[20,21]. There is a dearth of information within Nigeria regarding mental ill health morbidities as an aftermath effect of hysterectomy especially among those with benign clinical diagnosis. Therefore, this study aims to compare the pre and post hysterectomy mental ill health status as well as to document any association with their clinical indications.

## Methodology

This is a prospective study, conducted at the University College Hospital (UCH), Ibadan, Nigeria from January to June 2005. At UCH, three gynaecology clinics are held per week with an average of 25 to 50 patients per clinic comprising both new and follow-up patients. The routine practice at the clinic involves group health education about common gynaecological symptoms; measurement of vital signs and also counseling, clerking and examination by the attending doctor. The decision to have major surgery is usually taken by the consultant before pre-operative investigations are undertaken. Thereafter, the patient is then admitted for surgery at a scheduled date. The unit does not routinely involve clinical psychologists during the counseling session. The study was approved by the University of Ibadan/University of College Hospital Institutional Review Committee, Ibadan.

### Study Population

A convenient sampling of 50 consecutive participants scheduled for hysterectomy on account of benign clinical diagnosis at the Gynaecology outpatient clinic was recruited. A written consent was obtained from each participant before enrollment into the study. Those with malignant conditions, previous mental ill health or major surgeries - defined as any surgery that necessitated general anaesthesia, Sub-arachnoid or Epidural block were excluded as possible confounders.

### Survey Instrument

The mental health state was assessed using a validated 28-item General Health Questionnaire (GHQ), and this tool screened for anxiety and depression[22]. The score of less than 4 was interpreted to be normal while a score of 4 and above suggested mental ill health (psychiatric disorder). The instrument was interviewer administered using 2 trained research assistants. The pre-operative interview was performed 4 to 6 weeks before admission during which patients were either being investigated, pooling funds for surgery or awaiting admission. This period was selected to reduce the possibility of anxiety due to the surgery itself. The same questionnaire was also administered on the study population during the post-hysterectomy follow-up visit, usually 4 to 6 weeks after hospital discharge.

### Data Analysis

The data obtained was entered and analyzed using the Statistical Package for Social Sciences software 15.0. Information collected included biosocial variables such as the respondent's age, education, religion, marital status and parity as well as medical indication for the surgery and the grading of mental health states. Cross tabulations were performed to detect any association and also to compare pre and post hysterectomy mental health status. The level of statistical significance was set at P < 0.05.

## Results

Of the 50 women recruited, 5 declined to participate. The reasons for their decline were personal - 2, spousal's disapproval - 2 and no specific reason -1. The remaining 45 consented to participate in the study. The age of the women ranged from 35 to 63 years with a mean of 48.6 ± 0.6 years. The majority (93.3%) of the women was aged 40 years and above; with the age group 40-50 years constituting the largest group (51.1%)-see table 1. Married (95.5%) and women of Christian religious group (80.0%) were in the majority. Concerning their educational level, about two-thirds (64.4%) had tertiary education while others had secondary (22.0%), and primary or Koranic education (13.3%). Twenty (44.4%) of the study participants were unskilled workers; eighteen (40.0%) were professionals and the remaining seven (15.6%) were skilled workers. Most of the respondents had as many as 5 living children. Five had 6 or more children, while three had no living child. (Table 1). The indication for hysterectomy among the study population were uterine fibroids - 19, genital prolapse - 9, dysfunctional uterine bleeding - 6, and others (benign ovarian cyst, endometriosis, and adenomyosis) - 11 (Data not shown)

**Table 1:** Biosocial characteristics of the study participants (n = 45)

**Variables**	**Frequency**	**Percentage**
**Age**		
31 - 40	3	6.7
41 - 50	23	51.1
>50	19	42.2
**Religion**		
Christianity	36	80.0
Islam	7	15.6
Others	2	4.4
**Marital Status**		
Married	43	95.6
Widowed	2	4.4
**Educational level**		
Primary/Koranic	6	13.3
Secondary	10	22.0
Tertiary	29	64.4
**Occupation**		
Unskilled worker	20	44.4
Skilled worker	7	15.6
Professional	18	40.0
**No of living children**		
0	3	6.7
1-5	37	82.2
>6	5	11.1

On the pattern of mental ill health before hysterectomy, 20 (44.4%) had anxiety related disorders and 3 (6.7%) had depression. Post-hysterectomy, the number of those with anxiety related disorder increased to 22 (51.2%) while those with depression decreased to 2 (4.4%). Although, there was a proportional increase of 6.8 percent for anxiety related disorders and reduction of depression by 2.3 percent, the difference was not statistically significant. (Table 2).

**Table 2:** Pattern of Mental ill Health morbidities before and after hysterectomy

**Validated GHQ scores**	**Pre-hysterectomy** **N (%)**	**Post-hysterectomy** **N^P ^(%)**	**Changes**	**Remark**	**^F^p-value**
**Anxiety related disorder**					
Less than 4	25 (55.6)	21 (48.8)			
4 and above	20 (44.4)	22 (51.2)	6.8%	Increased	0.53
**Depression**					
Less than 4	42 (93.3)	41 (95.3)			
4 and above	3 (6.7)	2 (4.4)	2.3%	Reduced	0.69

GHQ - General Health Questionnaire

N^P ^- the post hysterectomy was 43.

^F^p - p-value using Fischer's exact test.

Regarding association between clinical indications for the hysterectomy and pattern of post operative mental ill health status, women with uterine fibroids had significant proportions of those with anxiety related disorders (68.4%) and depression (10.5%), while those grouped as "others" had participants with significant proportion of anxiety related disorders only (Table 3). Although, women that had genital prolapse and dysfunctional uterine bleeding as pre-operative diagnosis have 66.7 percent and 20 percent of anxiety related disorders respectively, however, the relationship is not of statistical significance.(Table 3).

**Table 3:** Relationship between post-operative mental ill health morbidities and indication for hysterectomy

**Surgical indication**	**Frequency**	**Anxiety related disorders**			**Depression**		
		
		GHQ score < 4 (%)	GHQ score >4 (%)	p value	GHQ score <4 (%)	GHQ score >4 (%)	p value
Uterine fibroid	19	6 (31.6)	13 (68.4)	0.00^s^	17 (89.5)	2 (10.5)	0.006^s^
Genital prolapse	9	3 (33.3)	6 (66.7)	0.12	9 (100.0)	Nil	
Dysfunctional Uterine bleeding	6	4 (80.0)	1 (20.0)	0.20	5 (100.0)	Nil	
Others	11	8 (80.0)	2 (20.0)	0.02^s^	10 (100.0)	Nil	

**Total**	**45**	**21**	**22**		**41**	**2**	

S - Statistically significant at p < 0.05

## Discussion

Uterine pathologies could signal the decision for its removal especially when the presenting symptoms have defied either expectant or medical treatment. Sometimes, patients often find it difficult to reconcile their life without their womb, and this makes their decision to consent for surgery challenging. Even after hysterectomy, evidence suggests that women still mourn the permanent loss of their uterus, and they sometimes feel the loss of their femininity[11].

In this study, the outcome shows a tendency towards increased anxiety related disorders and a reduction of depressive illness following hysterectomy. Though, this observed inference was not statistically significant as a result of small sample size. However, it is arguable from the available data that MIH could occur either before or after hysterectomy. The occurrence of anxiety related disorders and depression among the participants before hysterectomy is in tandem with studies from other settings[12,13,23] Suggested reasons for this change include hormonal effects, changes in physical appearances, being married or widowed, history of previous psychiatric disorders and poor sexual performance amongst others[9,24,25]. Although these reasons may have accounted for this cohort in this study, it is also probable that the higher anxiety related diagnosis recorded could be due to the general uncertainty that is often associated with the outcome of surgery in our environment. In addition, the non-availability of skilled professionals such as clinical psychologists that could dispel any concerns could be contributory.

Unlike report from elsewhere[14,26], though we recorded an increase in the proportion of those with anxiety related disorders by about 6.8 percent post- hysterectomy. Notwithstanding, the small sample size and the non-statistical significance of this observed pattern, the result could still offer some explanation in our setting. This finding may not be unconnected with the general lack of hormone replacement therapy among most Nigerian perimenopausal women that may reduce their psychosomatic symptoms such as emotional disturbances, anxiety and hot flushes. The lack is believed to be due to the high cost and non-availability. This peculiar challenge has led some Gynecologists within the country to advocate for ovarian conservation at hysterectomy in perimenopausal women to prevent earlier manifestation of psychosomatic and vasomotor symptoms. Expectedly, there was a significant reduction in the proportion of women with depression by 2.3 percent after hysterectomy. The plausible reason for this result may be due to the disappearance of their distressing preoperative complaints such as menorrhagia, disfiguring abdominal distension or chronic pelvic pain after the definitive care.

The fact that MIH still existed within the study population after the definitive hysterectomy raises concern about the possible association. This may suggest the possibility of a rethink from the opinion that hysterectomy is not associated with psychiatric disorder. In Nigeria, anecdotal evidence suggests that hysterectomised women may suffer emotional disturbance from the possible myth to matrimonial challenge. However, the exploration of the exact reasons from those noted with MIH will provide more insight in future research.

Like in many other previous studies[7,27], uterine fibroid forms the commonest indication for hysterectomy in this cohort. This finding further reaffirms the high prevalence of leiomyomata among Nigerians. Exploration of the relationship between preoperative clinical diagnosis and post-hysterectomy MIH revealed that those with uterine fibroids and "others" had significant proportion of anxiety related disorders. However, depression was only noticeable among the uterine fibroid subset. The possible reasons for this association may include the presenting symptoms, the myth/misconceptions about the aetiology, and fear of recurrence. It may be difficult to phantom any reason for the two women that had depression among those with fibroid because of their limited number. Genital prolapse is commoner in the elderly. Therefore, women with this diagnosis could have viewed their clinical condition as part of aging process, and thus cope better than others. Likewise, the disappearance of disturbing menorrhagia in those with Dysfunctional uterine bleeding might explain the lack of any significant association with post-hysterectomy MIH.

In conclusion, this study suggests that mental ill health may complicates hysterectomy for benign uterine pathology among Nigerian women, and that anxiety related disorders may increase after operation with the highest proportion in those with clinical diagnosis of uterine fibroid. We advocate preoperative psychological assessment as a preemptive measure, and possibly incorporate clinical psychologist counseling as part of preoperative care to minimize MIH among patients scheduled for hysterectomy. Future research on the peculiar socio-cultural implications, use of larger sample size and effect of hysterectomy will be a significant addition to the available evidence in Nigeria.

## Competing interests

The authors declare that they have no competing interests.

## Authors' contributions

OMA, UC and OOO coordinated the design, data collection and analysis of the Data; IOMB, OSN and OOA performed the literature search and eventual write-up of the manuscript. All authors read and approved the manuscript.
